# Identifying Qualitative Between-Subject and Within-Subject Variability: A Method for Clustering Regime-Switching Dynamics

**DOI:** 10.3389/fpsyg.2020.01136

**Published:** 2020-06-04

**Authors:** Lu Ou, Alejandro Andrade, Rosa A. Alberto, Arthur Bakker, Timo Bechger

**Affiliations:** ^1^ACTNext by ACT, Inc., Iowa City, IA, United States; ^2^Department of Mathematics, Freudenthal Institute, Utrecht University, Utrecht, Netherlands

**Keywords:** clustering, regime-switching model, functional data analysis, time-series data, dynamic model

## Abstract

Technological advancement provides an unprecedented amount of high-frequency data of human dynamic processes. In this paper, we introduce an approach for characterizing qualitative between and within-subject variability from quantitative changes in the multi-subject time-series data. We present the statistical model and examine the strengths and limitations of the approach in potential applications using Monte Carlo simulations. We illustrate its usage in characterizing clusters of dynamics with phase transitions with real-time hand movement data collected on an embodied learning platform designed to foster mathematical learning.

## 1. Introduction

Human dynamic processes vary within a subject over time and differ between subjects at all behavioral, physiological, emotional, attentional, and cognitive levels (Molenaar et al., 2003). Widespread examples include but not limited to change processes in belief and attitudes (van der Maas et al., 2003; Jansen et al., 2007), affective experiences (Cole et al., 2004; Kuppens et al., 2010; Hamaker et al., 2015), and executive functions (Zelazo, 2016). The within- and between-subject variabilities can be quantitative as well as qualitative in nature (Pintrich, 1988; Van Geert, 1991; van der Maas and Molenaar, 1992; van Dijk and van Geert, 2007; Stephen et al., 2009). For instance, human development is continuous and quantitative with gradual and incremental growth but simultaneously is discontinuous and qualitative as new forms and abilities emerge (Thelen and Smith, 1994). Inter-individual differences are also quantitative as no two individuals are identical within a population, and qualitative as subgroups of individuals may exist and share similar characteristics (Ram and Grimm, 2009; Bulteel et al., 2016). In order to understand the essence and drivers of human processes, researchers argue for a need to focus on studying and interpreting qualitative variability (Kelso, 2000). However, limited labor resources and subjectivity issues often put constraints on qualitative approaches (e.g., interviews and focus groups) that quest directly for qualitative findings. Alternatively, we infer qualitative changes and differences from data using quantitative methods that bring objectivity and computational accuracy and efficiency.

To this aim, we need mathematical and statistical models that represent both quantitative and qualitative within- and between-subject variability in the processes of interest and the data we collect. Mathematically, quantitative variability is often accommodated in continuous variables, while qualitative variability in categorical variables. The former refers to the within-subject numerical changes (including process noise) and between-subject random effects. In contrast, the latter refers to the within-subject regime (or phase) transitions and between-subject cluster (or group) differences. A cluster or group is a class of subjects that share similar qualities or dynamic patterns. A regime or phase is a within-subject time-varying class of dynamics that may switch from one to another as time passes. We use regime switches and phase transitions interchangeably. In our definitions, a regime or phase is different from a stage, which is a course in one-directional and non-reversible class transitions, such as age-based developmental stages.

Dynamic processes may exhibit qualitatively different cluster-wise quantitative changes interspersed with qualitative regime-switching. As an example, we consider students' learning processes that occur with dynamic sensorimotor coordination in an embodied learning environment. In a typical task, students acquire the concept of proportionality by coordinating movements of both their hands. Previous research found dynamic patterns and solution strategies from the patterns in students' action-coordination that relate to Piaget's theorized phases of reflective abstraction (Abrahamson et al., 2014; Duijzer et al., 2017; Pardos et al., 2018). The hand movements represent within-subject quantitative variability, and the strategies used form qualitatively switching regimes. High-performing and low-performing students may differ in the types and sequences of strategies used, thus displaying cluster-wise regime-switching patterns of hand movement dynamics. Hence, some interesting qualitative findings, in this case, are not only distinct regimes in students' strategy use or knowledge development but also clusters of regime-switching trajectories that are indicative of student's learning and have implications for interventions.

Many existing mathematical models only consider quantitative changes. For example, auto-regressive moving-average models and differential equations models represent quantitative within-subject changes in time-series data (Chow et al., 2005, 2011; Voelkle and Oud, 2013; Hu et al., 2014; Bulteel et al., 2016). The two types of modeling frameworks differ in whether the time in the model is discrete or continuous. They both are parametric models that exert top-down assumptions on the mechanism of change. In contrast, non-parametric methods like functional data analysis (Ramsay and Silverman, 2005) provide a bottom-up, data-driven way to approximate the dynamic changes directly using a combination of curves or smooth functions. Extensions and applications of these models allow for quantitative between-subject differences. For instance, when we apply these methods to a single subject's time-series data, we naturally allow each subject to have a unique set of parameters. When a law of change applies to the whole sample, we can include random effects that follow a specific statistical distribution to account for variability in model parameters (Oravecz et al., 2011; Lu et al., 2015; Chow et al., 2016; Ou et al., 2019).

Also, models that consider qualitative changes deal with clusters between subjects, or regimes within a subject but do not integrate the two. To capture qualitative between-subject variability, researchers use finite mixture models (McLachlan and Peel, 2004) to accommodate group differences by introducing a latent categorical variable that governs the emission of observed data. A finite mixture model assumes that a subject's data come from different latent groups with a particular set of probabilities. In each group, the emission of observed data follows different statistical distributions. In social and behavioral sciences, finite mixture models have been applied to identify latent groups with distinct means and covariance structures (Collins and Lanza, 2010), and factor structures (Lubke and Muthen, 2005; Hallquist and Wright, 2014). By incorporating assumptions on the longitudinal structure of quantitative changes, extensions of finite mixture models have been used to cluster subjects based on different growth trajectories (Colder et al., 2002; Muthen, 2004; Ram and Grimm, 2009), and dynamic emotional patterns in close relationships (Liu et al., under review).

Hidden Markov models are another standard class of models to analyze within-subject qualitative phase transitions. They have been widely applied in social and behavioral sciences to understand cognitive processes (Vermunt et al., 1999; Böckenholt, 2005; Dutilh et al., 2010; Visser, 2011; Visser and Speekenbrink, 2014; Andrade et al., 2017; Shu et al., 2017; Deonovic et al., 2018; Wang et al., 2018; Arieli-Attali et al., 2019). Hidden Markov models are extensions of the finite mixture models as the observed variables follow a mixture distribution depending on a latent categorical variable. The added feature is that the latent categorical variable can transition from one state to another in a first-order Markov chain, where the current state only depends on the previous state. Similar to finite mixture models, initial regimes and regime transitions are interpreted based on probabilities and the effects of covariates on these probabilities. As an extension of the hidden Markov model that considers longitudinal quantitative changes, regime-switching dynamic models permit modeling of manifest variables with discrete- or continuous-time equations rather than single emissions. Previous applications of the regime-switching models include the application of a regime-switching autoregressive model to facial Electromyography data to identify deactivated and activated emotional states (Yang and Chow, 2010), and the use of regime-switching differential equations to represent the regime transitions between exploration and proximity seeking of a child in mother-child interactions (Chow et al., 2018).

Despite the above developments, methods for simultaneously capturing both within- and between-subject qualitative variability (i.e., clusters and regimes) in time-series data with quantitative changes are nascent in social and behavioral sciences. In these fields, the quality of the data largely depends on the intrinsic complexity in human processes, the quality of measures (e.g., reliability and validity), and other economic, ecological, ethical, and privacy issues in data collection. As intensive longitudinal methods (Bolger and Laurenceau, 2013) become prevalent, an increasing number of data occur naturally at time points that are irregularly spaced within a subject and vary in the total number across subjects. Hence, data issues such as sample size (in terms of the number of subjects and number of measurements), noise, and missing data present challenges in applications of quantitative methods. In particular, while many clustering techniques require an equal dimension of data across subjects, data manipulation, including aggregation and imputation, is almost inevitable. Researchers that are interested in applying the methods need to understand whether the techniques are robust to the various data conditions they encounter and how the accuracy of the techniques varies with the data manipulation decisions that they have to make.

In this paper, we introduce and tailor an approach for characterizing qualitative between- and within-subject differences from quantitative changes to typical social and behavioral applications. We aim to present an elegant example for educational purposes and offer general guidance to researchers who wish to use the approach in their work. The approach is called the mixture of regressions with hidden logistic processes (mixRHLP; Chamroukhi et al., 2010, 2013; Samé et al., 2011), and was initially developed in engineering and science. It involves a complex but general modeling framework that integrates the finite mixture model for capturing group differences, a logistic regression model for explaining phase transitions, and a functional data analysis approach for non-parametrically representing the quantitative dynamics within a phase. We can estimate the mixRHLP model efficiently within the frequentist's framework. We are particularly interested in its strengths and limitations in understanding dynamic processes in social and behavioral sciences. Hence, we conduct Monte Carlo simulations to evaluate the performance of the approach and related model selection methods and test their robustness to various data limitations. We examine the fitting of the model to data with different sample sizes in terms of the number of subjects and the number of time points, proportions of missing values, and regression error variances. Then, we illustrate its usage by analyzing real-time hand movement data collected from an embodied learning platform designed to foster the learning of mathematical proportion. We offer practical guidance on data manipulation and model selection procedures based on the simulation results. Finally, we discuss the limitations, contributions, and future extensions of the current study.

## 2. Modeling Framework

The mixRHLP model (Chamroukhi et al., 2010, 2013; Samé et al., 2011) is designed to analyze multi-subject time-series data. Suppose for each subject *i, i* = 1, 2, 3, ⋯ , *N*_*p*_, there are a total of *N*_*t*_ measurement occasions and *N*_*t*_ measurements of an interesting process (e.g., sensory data of student behavior and emotion), respectively denoted as *N*_*t*_ × 1 vectors of ***t*** = (*t*_*j*_) and ***y***_*i*_ = (*y*_*i*_(*t*_*j*_)). *j* = 1, 2, 3, ⋯ , *N*_*t*_ indexes *N*_*t*_ measurement occasions, and (*t*_*j*_) is a set of continuous values that indicate elapsed time since each subject's onset and stay the same for all subjects. Thus, ***t*** = (*t*_*j*_) represents a shared time frame for all subjects, whereas ***y***_*i*_ = (*y*_*i*_(*t*_*j*_)) exhibit variability across subjects and over time. We assume ***y***_*i*_ follow a mixture distribution, whose density *p*(·) is a weighted sum of component densities *p*_*k*_(·) as

(1) $$\begin{array}{l} p\left(y_{i}|t_{i};\Theta \right)=\overset{K}{\underset{k}{\sum }}P\left(Z_{i}=k\right)p_{k}\left(y_{i}|t_{i},Z_{i}=k\right), \end{array}$$

where *Z*_*i*_ ∈ {1, 2, ⋯ , *K*} denotes subject *i*'s latent cluster class, with $\alpha_{ik}\overset{\Delta }{=}P\left(Z_{i}=k\right)$ being the probability of subject *i* belonging to the latent cluster class *k*. **Θ_*k*_** contains all parameters in the component density *p*_*k*_(·), and **Θ** contains all parameters in the density *p*(·).

At each time point *t*_*j*_, we further assume *y*_*i*_(*t*_*j*_) follows a finite Gaussian mixture regression model, whose conditional component density given cluster *k* and regime *r* = 1, 2, ⋯ , *R* is normally distributed with mean ***X***_*j*_**β**_*kr*_ and a variance of $\sigma_{kr}^{2}$, denoted as $N\left(X_{j}\beta_{kr},\sigma_{kr}^{2}\right)$. That is, in each regime, the temporal dynamics of ***y***_*i*_(·) is captured by a linear regression model of time. While the design matrix in the regression model may take different forms, we assume that the regression model is a polynomial regression model of order *d*, where the design matrix ***X***_*j*_ is $\left[\begin{array}{cccc} t_{j}^{0} & t_{j}^{1} & \cdots & t_{j}^{d} \end{array}\right]$ and **β**_*kr*_ is a (*d* + 1) × 1 vector of regression coefficients ${\left[\begin{array}{ccc} \beta_{kr0} & \beta_{kr1} & \cdots \beta_{krd} \end{array}\right]}^{⊤}$. If we further assume ***y***_*i*_|***t***_*i*_, *Z*_*i*_ = *k* given subject *i*'s latent cluster class *Z*_*i*_ = *k* are serially independent, then the component density *p*_*k*_(·) can be written as

(2) $$\begin{array}{ll} p_{k}\left(y_{i}|t_{i},Z_{i}=k\right) & =\overset{N_{t}}{\underset{j=1}{\prod }}\overset{R}{\underset{r}{\sum }}P\left(H_{ij}=r|t_{j},Z_{i}=k\right)N\left(X_{j}\beta_{kr},\sigma_{kr}^{2}\right), \end{array}$$

where *H*_*ij*_ ∈ {1, 2, ⋯ , *R*} denotes subject *i*'s latent regime at time *t*_*j*_ and takes categorical values of {1, 2, ⋯ , *R*}.

The latent regime *H*_*ij*_ at each time point *t*_*j*_ is assumed to follow a multinomial logistic regression model such that the probability of subject *i* belonging to the latent regime *r* at time *t*_*j*_ under the condition that subject *i* belongs to the latent cluster class *k* is

(3) $$\begin{array}{l} P\left(H_{ij}=r|t_{j},Z_{i}=k\right)=\frac{\exp\left(\omega_{kr0}+\omega_{kr1}t_{j}\right)}{\sum_{s=1}^{R}\exp\left(\omega_{ks0}+\omega_{ks1}t_{j}\right)} \end{array}$$

with ω_*ks*0_ = ω_*ks*1_ = 0 in a reference class. The regression coefficients **ω**_*kr*_ = [ω_*kr*0_ ω_*kr*1_] control the regime switches, and thus are regime-switching parameters. For instance, if *R* is the reference class, ω_*kR*0_ = ω_*kR*1_ = 0. Then, ω_*kr*0_ + ω_*kr*1_*t*_*j*_ is the log-odds or relative probability of subject *i* belonging to regime *r* at time *t*_*j*_ compared to the reference regime *R*, given that the subject is in cluster *k*. In the log-odds, ω_*kr*0_ is an intercept and ω_*kr*1_ is a slope. If ω_*kr*1_ is positive, this relative probability increases over time. Hence, if the probability of being in the reference regime *R* stays the same across time, a positive ω_*kr*1_ indicates that the likelihood of being in regime *r* goes up with time. In this way, these parameters influence regime switches.

Assuming the observed data $Y\overset{\Delta }{=}\left[y_{i}\right]$ across subjects are independently identically distributed, we can write the log-likelihood function of **Θ** given all observed data as

(4) $$\begin{array}{l} l\left(\Theta \right)=\log\overset{N_{p}}{\underset{i}{\prod }}p\left(y_{i}|t_{i};\Theta \right)=\overset{N_{p}}{\underset{i}{\sum }}\log\overset{K}{\underset{k}{\sum }}\alpha_{ik}p_{k}\left(y_{i}|t_{i},Z_{i}=k\right). \end{array}$$

Parameter estimation can be obtained via the Expectation-Maximization algorithm (Dempster et al., 1977). To evaluate the quality of the model, we use the following information criteria: Bayesian Information Criterion (BIC; Schwarz, 1978), sample-adjusted BIC (saBIC; Sclove, 1987), the Akaike Information Criterion (AIC; Akaike, 1973) and the corrected AIC (AICc; Hurvich and Tsai, 1989) for model selection. Each criterion is defined by the difference between the maximized log-likelihood *l*_*M*_(**Θ**), and a penalty score based on the number of parameters |**Θ**|, and weights goodness of fit against model simplicity: *BIC* = log(*N*_*t*_ × *N*_*p*_)|**Θ**| − 2*l*_*M*_(**Θ**), $saBIC=\log\left(\frac{N_{t}\times N_{p}+2}{24}\right)|\Theta |-2l_{M}\left(\Theta \right)$, *AIC* = 2|**Θ**|−2*l*_*M*_(**Θ**), and $AICc=AIC+\frac{2|\Theta {|}^{2}+2|\Theta |}{N_{t}\times N_{p}-|\Theta |-1}$. The model yielding the lowest criterion value is perceived as the model that generalizes best (Myung and Pitt, 2018).

The estimation algorithm also computes the posterior regime and cluster probabilities at each time point as by-products. We can determine cluster and regime classifications by the highest posterior probability in posterior class probabilities at each time point.

## 3. Simulation

### 3.1. Simulation Design

As many naturally collected data contain a small sample size and are collected at irregular intervals, we conducted Monte Carlo simulations to evaluate the applicability of the mixRHLP model under these limitations. In particular, we were interested in (1) whether the information criteria could be useful in model selection, (2) how accurate the estimation algorithm could be in estimating parameters and making classifications, and (3) how the answers to (1) and (2) would change under different data conditions. We sought to examine the fitting of the model to data with different sample sizes in terms of the number of participants and the number of time points, proportions of missing values, and regression error variances.

**Figure 1:**
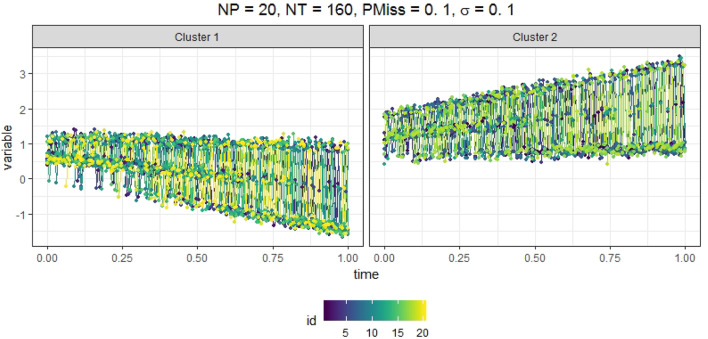
Simulated time series in two clusters when *N*_*p*_ = 20, *N*_*t*_ = 160, *PMiss* = 0.1, and σ = 0.1.

We generated data from a mixRHLP model with 2 clusters (*K* = 2), 3 regimes (*R* = 3), and linear functions (*d* = 1). We wanted the K, R, d values to be as small as possible so that the model is simple enough but still exhibits minimal cluster-based regime-switching properties with time-dependent structure in each regime. We chose *R* = 3 instead of 2 to mirror the regime characteristics observed in our empirical data. The measurement occasions ***t*** were equally spaced time points within the interval of [0, 1]. The true parameter values are listed in Table 1 and were selected such that in different clusters and regimes the dynamics varied but were hard to differentiate by eyes when plotted altogether. We assumed equal regression error variance σ across clusters and regimes, and that the data may be missing completely at random. We varied four factors in simulating the data: (1) the number of participants in the sample (*N*_*p*_ = 20, 60, 100), (2) the number of time points (*N*_*t*_ = 20, 160, 300), (3) the magnitude of the regression error variance (σ = 0.10, 0.15, 0.20), and (4) the proportion of missing data in each participant's data (*PMiss* = 0, 0.1, 0.2). Figure 1 showed the simulated data in two clusters under the conditions of *N*_*p*_ = 20, *N*_*t*_ = 160, *PMiss* = 0.1, and σ = 0.1.

**Table 1:** True parameter values used in the Monte Carlo simulation study.

			**Regime 1**		**Regime 2**		**Regime 3**	
		**X**	**1**	**t**	**1**	**t**	**1**	**t**
Cluster 1	α_1_ = 0.5	**ω**_1·_	−2.00	3.00	1.00	−2.50	0	0
		**β**_1·_	0	−1.50	0.60	−0.90	1.20	−0.30
		σ_1_	0.10, 0.15, 0.20					
Cluster 2	1−α_1_ = 0.5	**ω**_2·_	-1.00	2.00	0.50	−2.00	0	0
		**β**_2·_	0.60	0.30	1.20	0.90	1.80	1.50
		σ_2_	0.10, 0.15, 0.20					

We carried out *M* = 200 Monte Carlo runs for each of the 81 (= 3^4^) data conditions. Where data were missing for a participant, we replaced the missing values using linear interpolation with the *na.approx()* function from the **zoo** R package (Zeileis and Grothendieck, 2005). To each set of full data (after imputation), we fitted a total of 32 mixRHLP models with combinations of different values of *K* = 1, 2, 3, 4, *R* = 1, 2, 3, 4, and *d* = 1, 2 and heteroskedastic regression error variances using the **mixRHLP** package (Chamroukhi et al., 2010, 2013; Samé et al., 2011). When the algorithm finished successfully, we computed the four information criteria: BIC, saBIC, AIC, and AICc.

We used three sets of measures to compare the model fitting results across simulation conditions: (1) information criteria measures, (2) parameter estimate accuracy measures and (3) classification accuracy measures. Information criteria measures included a proportion measure and a rank measure. The proportion measure is the proportion of runs where a certain criterion of the true model (*K* = 2, *R* = 3, *d* = 1) indicated itself as the best-fitting model (i.e., as the smallest among those of the 32 fitted models). The rank measure is the average rank of the criterion value among ordered values of the 32 models' same criterion arranged from the smallest to the largest. To measure parameter estimate accuracy, we computed the root mean squared errors of each parameter. To simplify the presentation of the simulation results, we grouped the parameters into six sub-groups, namely, α_1_, **β**_0_ = [β_*kr*0_], **β**_1_ = [β_*kr*1_], σ, **ω**_0_ = [ω_*kr*0_], and **ω**_1_ = [ω_*kr*1_] and took the average RMSE of the parameters within the same sub-group. Let θ_*g, G*_ and ${\hat{\theta }}_{r,g,G}$ respectively denote the true and estimated value of a parameter, where *r* indicates the *r*-th Monte Carlo run, and *g* indicates the *g*-th parameter in a parameter group *G* of size |*G*|. The average RMSE was computed as ${\text{rmse}}_{\text{G}}=\frac{1}{\left|\text{G}\right|}\underset{g}{\sum }\sqrt{\frac{1}{M}\underset{r}{\sum }{\left({\hat{\theta }}_{r,g,\text{G}}-\theta_{g,\text{G}}\right)}^{2}}$. The classification accuracy measures are the proportion of correct classifications of either the clusters or the regimes of available data (before imputation).

### 3.2. Simulation Results

To reveal the typical characteristics of the Monte Carlo samples, we decided to remove the outliers of the simulation measures within each data condition. We used the *OutlierDetection()* function in the R package **OutlierDetection** (Tiwari and Kashikar, 2019) to identify outliers based on K-nearest neighbor graphs (K = 5% of the Monte Carlo runs, Hautamaki et al., 2004). The remaining Monte Carlo sample size ranged from 165 to 197, with a median of 191. Most outliers were found when the sample size was the smallest (*N*_*p*_ = 20, *N*_*t*_ = 20), regression error variance high (σ = 0.2), and missing data imputation involved (*PMiss* = 0.1).

Among the rest of the Monte Carlo samples after outlier removal, BIC performed better than the other three information criteria in selecting the right model as the best-fitting model, with a success rate of 0.54, whereas the success rates of saBIC, AICc, AIC were 0.38, 0.21, and 0.20, respectively. The median rank of the true model's BIC among 32 models was 1 (i.e., the smallest), and the maximum rank was 10, both smaller than those of saBIC (median 3, maximum 12), AIC (median 5, maximum 12) and AICc (median 5, maximum 12). Although BIC could be useful for model selection under certain conditions, the smallest BIC did not always indicate the true model in simulations. When we fitted the correct model, the accuracy of the parameter estimates was high, characterized by RMSEs lower than 0.1, except for the regime-switching parameters in **ω**_0_ and **ω**_1_ categories. Even though some of the regime-switching parameters could not be estimated correctly, the classification accuracy was overall very high. Across all data conditions, the proportion of correct cluster classifications was invariably 1, and the proportion of correct regime classifications was 0.99, suggesting the robustness of the approach in identifying clusters and regimes in time-series data of our interest.

**Figure 2:**
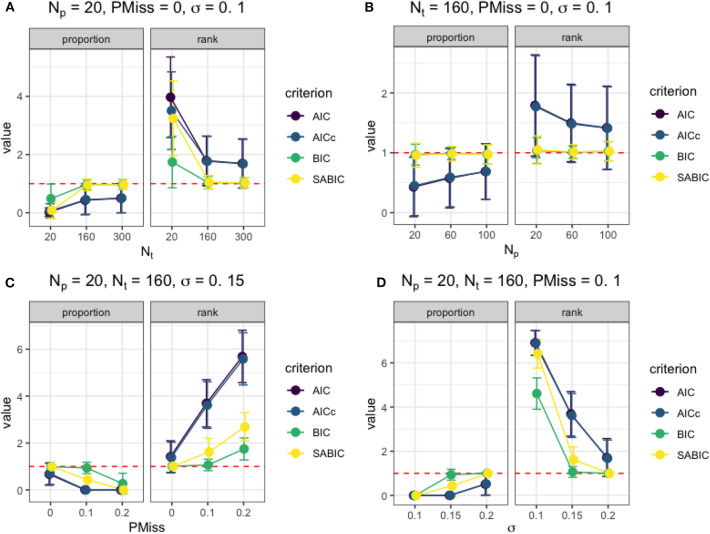
The utility of information criteria under different simulation conditions. **(A)** The effect of *N*_*t*_ on the information criteria measures when *N*_*p*_ = 20, *PMiss* = 0, σ = 0.1. **(B)** The effect of *N*_*p*_ on the information criteria measures when *N*_*t*_ = 160, *PMiss* = 0, σ = 0.1. **(C)** The effect of *PMiss* on the information criteria measures when *N*_*p*_ = 20, *N*_*t*_ = 160, σ = 0.15. **(D)** The effect of σ on the information criteria measures when *N*_*p*_ = 20, *N*_*t*_ = 160, *PMiss* = 0.1.

After examining the results from each simulation condition, we identified how the four factors considered affected the model selection and statistical inference. Figure 2 presents the effects of the factors on the information criteria measures under typical simulation conditions. When the data were at a sufficient number of time points (e.g., *N*_*t*_ ≥ 160) and without missing data, the smallest BIC could be used to select the correct model as the best-fitting model regardless of *N*_*p*_ and σ. As shown in Figure 2A, the BIC and saBIC of the true model were almost always the smallest among fitted models when *N*_*t*_ was higher than 160, and there was no missing data. Also, the utility of information criteria improved with an increase in *N*_*t*_ even though *N*_*p*_ was small, with higher success rates in selecting the correct model and lower rank among fitted models. When *N*_*t*_ = 160 under the same condition without missing data (e.g., in Figure 2B), although BIC and saBIC performed almost equally well, the utility of AIC and AICc improved as *N*_*p*_ increased. However, when the imputation of missing data happened, the larger the size of the missing data, either as a result of a bigger sample size or a more substantial missing proportion (e.g., partly illustrated in Figure 2C), the smaller the utility of all information criteria was. Nevertheless, when the regression error variance in the actual model was high, the misfit of the mixRHLP model to imputed data could be considered as regression errors, enabling the use of information criteria in model selection, as shown in Figure 2D.

**Figure 3:**
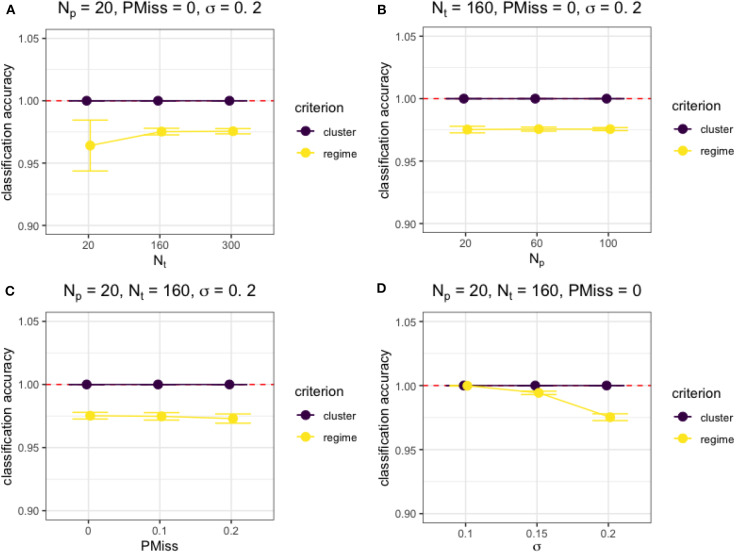
The accuracy of classification under different simulation conditions. **(A)** The effect of *N*_*t*_ on the cluster and regime classification accuracy measures when *N*_*p*_ = 20, *PMiss* = 0, σ = 0.2. **(B)** The effect of *N*_*p*_ on the cluster and regime classification accuracy measures when *N*_*t*_ = 160, *PMiss* = 0, σ = 0.2. **(C)** The effect of *PMiss* on the cluster and regime classification accuracy measures when *N*_*p*_ = 20, *N*_*t*_ = 160, σ = 0.2. **(D)** The effect of σ on the cluster and regime classification accuracy measures when *N*_*p*_ = 20, *N*_*t*_ = 160, *PMiss* = 0.

Besides, Figure 3 shows the effects of the four factors on the classification accuracy measures under typical simulation conditions. Generally, both the cluster and regime classifications were accurate and not affected by sample size (*N*_*p*_ or *N*_*t*_) nor proportion of missing data (*PMiss*), unless the sample size was really small (i.e., *N*_*t*_ = *N*_*p*_ = 20) and the regression error variance was high, as shown in Figures 3A,D. However, the regime classification accuracy depended on the characteristics of the model. For example, the larger the regression error variance was, the lower the accuracy of regime classifications (see Figure 3D).

**Figure 4:**
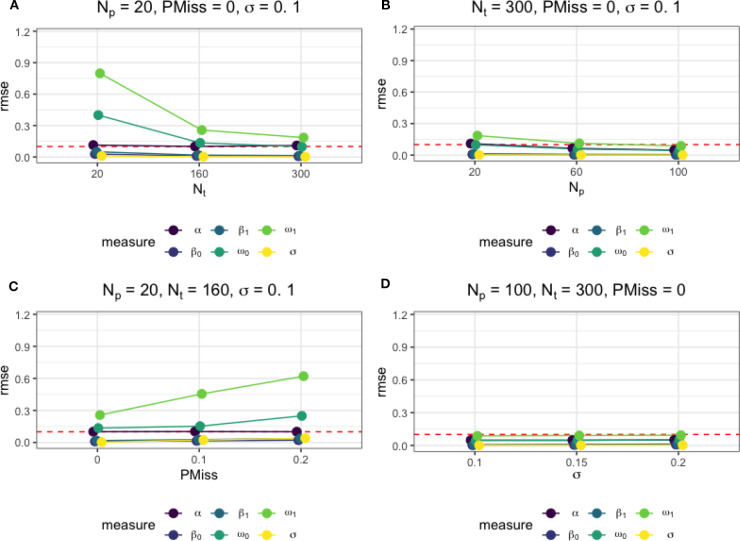
The average root mean square error (RMSE) of parameter estimates under different simulation conditions. **(A)** The effect of *N*_*t*_ on the parameter estimate accuracy measures when *N*_*p*_ = 20, *PMiss* = 0, σ = 0.1. **(B)** The effect of *N*_*p*_ on the parameter estimate accuracy measures when *N*_*t*_ = 300, *PMiss* = 0, σ = 0.1. **(C)** The effect of *PMiss* on the parameter estimate accuracy measures when *N*_*p*_ = 20, *N*_*t*_ = 160, σ = 0.1. **(D)** The effect of σ on the parameter estimate accuracy measures when *N*_*p*_ = 100, *N*_*t*_ = 300, *PMiss* = 0.

Moreover, Figure 4 presents how different factors affected the accuracy of the estimates of the regime-switching parameters. As in Figures 4A,B, the larger the sample size was, as a result of an increase in either *N*_*t*_ or *N*_*p*_, the more accurate the parameter estimates. When there were no missing data, a sample of size *N*_*p*_ = 100 and *N*_*t*_ = 300 was sufficient for accurate estimation of all model parameters, with the RMSEs below a threshold of 0.1. The magnitude of regression error variance did not affect the accuracy parameter estimates, as seen in Figure 4D. However, as in Figure 4C, the presence of missing data, although imputed, affected the parameter estimation negatively. An increase in the proportion of the missing data led to higher RMSEs of the regime-switching parameters. In Figure 4C, we also present the RMSEs of the parameters under the data condition that is close to the data in our empirical example.

## 4. Empirical Example

**Figure 5:**
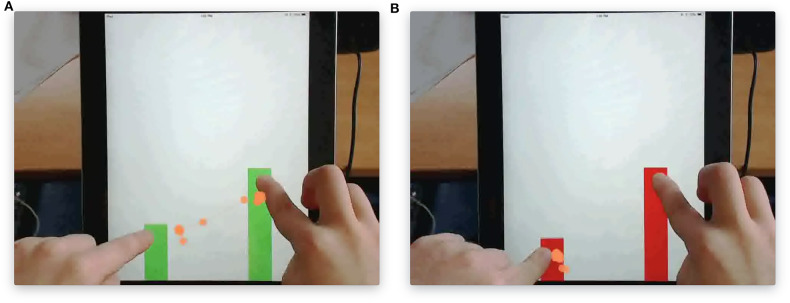
The touchscreen tablet version of the Mathematical Imagery Trainer for Proportion (MIT-P). **(A)** Fingers maintain a 1:2 ratio to make the bars green. **(B)** Fingers do not maintain such ratio and therefore the bars are red.

To illustrate our approach with real data, we built upon the work of the Mathematics Imagery Training of Proportion (MIT-P) and analyzed secondary data collected from a previous study (Abrahamson et al., 2015; Duijzer et al., 2017) with informed consent from the legal guardians of the participants and approval of the ethical committee board of the faculty of Social Sciences at Utrecht University. In the study, 45 fifth- and sixth- graders of ages 9–11 participated in task-based semi-structured interviews at schools in the Netherlands. In the interview, the participants played with a touchscreen tablet and used their index fingers to move two parallel vertical bars up and down (see Figure 5). The bars changed colors between red and green based on their heights. The closer the ratio between the height of right and left bars was to a predefined value (1:2), the greener the bars were, which was the mysterious rule the participants did not know before the interview and needed to find out. In the beginning, the participants were given instructions to move the bars and *find as many greens as possible*. After they found the first green, the participants were encouraged to find *more*. In the end, the participants needed to *move the bars from the bottom to the top while keeping them green*. During the process, participants were probed to think aloud *why the bars turned green and what actions they were to take to solve the problem*. The same procedures applied under different task and screen conditions, where the proportional value varied from $\frac{1}{2}$ to $\frac{3}{4}$ or grids with and without numbers appeared on the screen. Screen recordings of participants' hand movements, together with tracking of their eye movements and concurrent verbalization, were captured during the whole interview. The data of 38 participants were of sufficiently high quality to include them in the analysis. The mean age of the participants was 11.3 years old (*SD* = 0.70), and there were 17 females in the sample. For retaining time series data under the same task and screen condition from all participants, we only focused on hand movement data collected in the task with the proportion of 1:2 and on blank screen background without grids.

**Figure 6:**
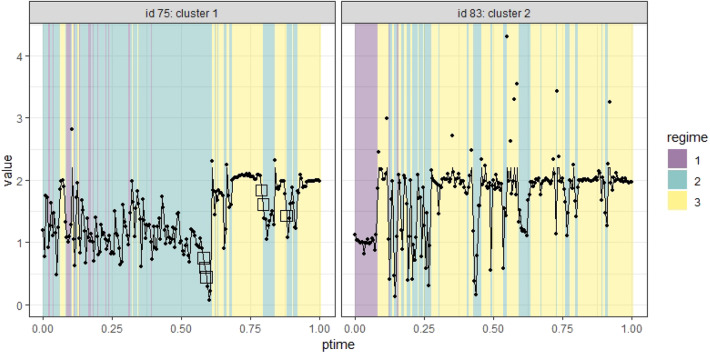
Time series data of two individuals identified in two clusters. The imputed data are marked as squares.

The original real-time capture of hand movement data happened as the participants moved their fingers on the tablet, and hence the data contained missing values and were irregularly spaced. To prepare the data for our analysis, we first removed data with a partial recording of only one hand's movement, which took up <5% of the available data and was missing largely because of off-task behavior and technical errors. The remaining data for each participant varied in the number of measurement occasions (6,132–32,543) and the total period they covered (3.28–13.71 min, with a mean of 6.74). To construct a common time frame for all participants, we re-scaled individuals' measurement occasions to a range of [0, 1] by subtracting the initial time point and dividing the times by the total period of each individual. We then aggregated data at the individual level in 200 equally spaced intervals in [0, 1) using their mean to create a data set of 38 participants on the same 201 occasions equally spaced in [0, 1]. In cases where there was no recording in a certain time interval for an individual, missing data would occur in the aggregation. In the new data, the proportion of missing data ranged from 0 to 0.17, with a median of 0.05, across individuals. We replaced the missing values with linear interpolation via the *na.approx()* function in the R package **zoo**. We took the ratios between right and left-hand positions as our variable of interest and winsorized the data by substituting the extreme ratios that are above the 95 percentile of the ratios with the 95 percentile. Figure 6 shows the aggregated time series of two individuals in points, and the imputed and winsorized data in lines. We marked the imputed data with squares.

We fitted the mixRHLP models to the time series data with different values of *K* (1–4), *R* (1–4), and *d* (1–2). Among all 32 models, we chose the more parsimonious model with the top three smallest BIC values, which consisted of two clusters, three regimes, and linear regressions. The parameter estimates from fitting the chosen model to the data are summarized in Table 2. The probability of an individual being in Cluster 1 was estimated to be 0.42, a little smaller than that of being in Cluster 2. Although the regimes' regression parameters differed across clusters, the respective three regimes were comparable in the two clusters. In particular, Regime 1 in both clusters had regression intercepts near one with small error variances, indicating hands were moving at the same height. Regime 2 in both clusters had significant error variances with intercepts around one, indicating hands moving with a noticeable variability. Regime 3 in both clusters had intercepts about two with small error variances, suggesting hands moving at the desired heights of 1:2 ratio to keep the bars green.

**Table 2:** Parameter estimates from the empirical example.

			**Regime 1**		**Regime 2**		**Regime 3**	
		**X**	**1**	**t**	**1**	**t**	**1**	**t**
cluster 1	α_1_ = 0.42	**ω**_1·_	1.899	−7.871	1.864	−2.597	0	0
		**β**_1·_	1.014	−0.019	1.149	0.016	2.017	−0.061
		$\sigma_{1}^{2}$	0.003		0.180		0.015	
cluster 2	1−α_1_ = 0.58	**ω**_2·_	1.423	−14.651	0	0	−0.386	1.794
		**β**_2·_	1.018	−0.039	0.896	0.594	2.027	−0.043
		$\sigma_{2}^{2}$	0.009		0.213		0.009	

After completing our statistical analysis, we also wanted a qualitative interpretation of the results in light of the possible solution strategies subjects were following during each regime. Accordingly, Regime 1 corresponded to an initial phase of the embodied interaction. During this regime, the hands were at the same height; perhaps the student awaited to see what happened next. Regime 2 corresponded to an intermediate phase of the interaction. During this regime, it seems as though the participant was actively exploring different hand ratios, perhaps attempting to find how to make the bars green. From prior qualitative observations, we know that this regime contains a mixture of strategies in that changes in the hands' ratio not only happens when the two hands move independently but also when they move at fixed distances. As our analysis missed this distinction, this seems to be one of the limitations of our current approach. Regime 3 corresponded to a later phase of the interaction and was the desired outcome of the interview. During this phase, the hands maintained a 1:2 ratio. However, as the task asked students to find green in as many ways as they could, from time to time, this particular ratio was lost, and the student fell back into Regime 2. Note that to keep the same ratio as the hands move up, the one hand has to move twice as fast as the other hand, which proves to be a challenging bodily coordination exercise for participants even though they have figured out the proportion rule. Further analysis of the participants' verbalization during the interview using natural language processing techniques confirmed our interpretation of the different regimes to some extent [see Ou et al. (2020) for more details].

**Figure 7:**
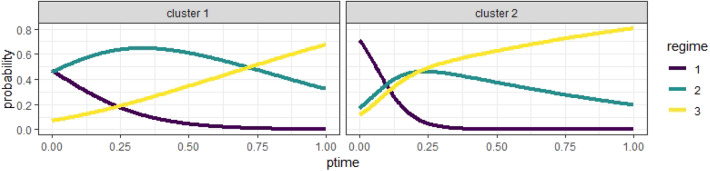
The estimated logistic curves of different regimes in two clusters.

Additionally, Figure 7 illustrates the estimated expected logistic curves of the probabilities of an individual being in a regime during the interview. In Cluster 1, the probability of being in Regime 1 was the highest at the start of the session but close to the probability of being in Regime 2, which grew slowly but soon became the highest until Regime 3 became the most probable regime at around 70% into the interview session. In Cluster 2, the probability of being in Regime 1 was the highest until approximately 10% into the session, when the probability of being in Regime 2 took the lead but was only slightly higher than that of being in Regime 3; then, Regime 3 became the most probable state at about 20% into the session, much sooner compared to Cluster 1. Indeed, what the logistic curves tell us is that a student in Cluster 1 has about the same likelihood to find the rule than not to find it, as indicated by the high logistic curve of Regime 2 for most of the task segment. Instead, students in Cluster 2 have a much higher probability of finding the proportional rule, especially after the first half. It is apparent that, in Cluster 2, the probability of being in Regime 1 goes down a lot more quickly than that in Cluster 1, and almost disappears after the first quarter. On the other hand, the probability of Regime 2 goes down but still lingers on, albeit low, until the end of the task segment.

To exemplify these results, Figure 6 shows different hand movement dynamics of participants with IDs 75 and 83. Participant 75, classified in Cluster 1, spent a substantial proportion (> 50%) of the session exploring various ratios (Regime 2) or merely moving her hands at the same speed (Regime 1). Participant 83, classified in Cluster 2, spent only 10% of the time moving hands at the same heights (Regime 1) and quickly switched to a 1:2 ratio phase (Regime 3), interspersed with chunks of short periods of Regime 2.

## 5. Limitations

The modeling framework and our empirical illustration have some limitations. First, a shared time frame of measurement occasions needs to apply to all subjects, which is often unrealistic in data collection. As participants differed in their time spent on the task, we were only able to construct a proportional time relative to their respective elapsed time such that the time frame is within [0, 1]. Besides, we had to involve data aggregation and missing data imputation based on the shared time frame, which could affect the accuracy of parameter estimates.

Second, the modeling framework and estimation algorithm only apply to univariate time-series data at this moment. Our example only took into account the hands' ratio, so we were not able to identify from the ratio data some of the strategies discussed in prior studies, such as the fixed-distance strategy with which participants kept their hands at the same speed. We could utilize eye gaze data and other hand-movement variables such as speed and distance between hands to study how hand and eye movements coordinate in such activities.

Third, the logistic transition process in Equation (3) assumes that the log odds of being in a regime relative to the reference regime change monotonically with time. It ignores the local context of a regime switch such as the current regime from which a switch is happening. Further, it lacks some flexibility in modeling bidirectional regime switches that are more common in hidden Markov type models and may apply under different circumstances.

Despite the limitations, the mixRHLP model is useful in extracting qualitative clusters and regimes from quantitative time-series data, and the illustrative example furthers our knowledge of qualitative differences in how students approach the mathematical concept of proportion physically.

## 6. Discussion

Advancements in real-time data capture technology revolutionized the type and amount of data we collect about human dynamic processes. In this paper, we have introduced the mixRHLP model for clustering multi-subject time-series data with regime-switching properties. In a Monte Carlo simulation study, we examined the accuracy of the approach in parameter estimation and cluster and regime classification under various data conditions. We tested the feasibility of using information criteria for model selection. We showed how different factors such as the number of time points, the number of participants, the proportion of missing data, and the error variance in the model could affect the performance and applicability of the approach and had a deeper understanding of the strengths and limitations of the approach.

To illustrate the use of this approach in real scenarios, we applied it to studying students' behavior in an action-based learning environment for mathematical learning. We based our data aggregation and model selection decisions on the Monte Carlo simulation results. We discovered qualitative differences in students' hand movements on a tablet during the task and across students, as they explored the concept of proportion using physical actions. This type of analysis helped reveal between and within-subject differences in dynamic processes not seen with prior qualitative analyses (Duijzer et al., 2017). That is, although qualitative analysis may help reveal phase transitions in strategy use, efficiently comparing students' experiences and performing grouping exceeded human capacity. Using the approach, we can not only extract strategies directly and efficiently from data but also identify clusters of students with homogeneous dynamics and potentially similar needs for intervention.

In the future, we should extend the estimation algorithm to fit multivariate time series data to account for systematic changes in dynamic systems. For instance, Kuppens et al. (2007) found that the extent to which individuals experience qualitatively different feelings in the core affect space is a consistent measure to their trait measures of self-esteem and depression. We need cluster-based multivariate dynamic models potentially with regime-switching features to help reveal systematic emotion dynamics that may have implications for psychological well-being and adjustment. Besides, we need to compare the mixRHLP modeling approach to other model-based and data-driven approaches for clustering regime-switching dynamics in simulations and applications. Candidate approaches include but not limited to the mixture of hidden Markov models (Chamroukhi and Nguyen, 2019) and potential extensions of existing data-driven methods that identify clusters or regimes (e.g., Cabrieto et al., 2018). Moreover, it is worthwhile to examine different imputation methods for missing data, for example, the newly developed ones that depend on machine learning approaches (Yoon et al., 2018).

Finally, the model contributes to the tools to extract qualitative cluster and regime patterns from quantitative time-series of human dynamics. We anticipate its broader usage in analyzing the increasingly prevalent multi-modal time-series data in social and behavioral sciences beyond mathematics learning. In applications, developers and practitioners may use the qualitative findings from time-series data to inform intervention and training programs. For instance, in collaborative learning environments such as classrooms, we might be able to monitor students' real-time behavior with various sensors and utilize the technique to generate learners' qualitative profiles and tailor personalized or group-based feedback to facilitate learning and shift students from one cluster to another.

## Data Availability Statement

The datasets generated for this study are available on request to the corresponding author.

## Ethics Statement

The studies involving human participants were reviewed and approved by the ethical committee board of the Faculty of Social Sciences at Utrecht University. Written informed consent to participate in this study was provided by the participants' legal guardian or next of kin.

## Author Contributions

LO worked on simulations and data analysis and prepared the first draft. RA and AB provided the raw data. All authors contributed to writing and editing the paper.

## Conflict of Interest

LO, AA, and TB were employed by the non-profit company ACT, Inc. The remaining authors declare that the research was conducted in the absence of any commercial or financial relationships that could be construed as a potential conflict of interest.
